# Epigenetic regulation of matrix metalloproteinase expression in ameloblastoma

**DOI:** 10.1186/1472-6890-12-11

**Published:** 2012-08-06

**Authors:** Lucyana Conceição Farias, Carolina Cavaliéri Gomes, Marcela Carolina Rodrigues, Wagner Henriques de Castro, Júlio César Tanos Lacerda, Efigênia Ferreira e Ferreira, Ricardo Santiago Gomez

**Affiliations:** 1 Department of Oral Surgery and Pathology, School of Dentistry, Universidade Federal de Minas Gerais, Av. Antonio Carlos, 6627, CEP 31270 901 , Belo Horizonte, Minas Gerais, Brazil; 2 Department of Pathology, Biological Sciences Institute, Universidade Federal de Minas Gerais, Belo Horizonte, Brazil; 3 Department of Oral Surgery and Diagnosis, Odilon Behrens Hospital, Belo Horizonte, Brazil; 4 Department of Social and Preventive Dentistry, School of Dentistry, Universidade Federal de Minas Gerais, Belo Horizonte, Brazil

**Keywords:** Ameloblastoma, Odontogenic tumours, Matrix metalloproteinases, MMP-2, MMP-9, Methylation, Epigenetic

## Abstract

**Background:**

An ameloblastoma is a benign odontogenic neoplasm with aggressive behaviour and high recurrence rates. The increased expression of matrix metalloproteinases (MMPs) has been reported in ameloblastomas. In the present study, we hypothesised that epigenetic alterations may regulate *MMP* expression in ameloblastomas.

**Methods:**

We investigated the methylation status of the genes *MMP-2* and *MMP-9* in addition to mRNA transcription and protein expression in ameloblastomas. Methylation analysis was performed by both methylation-specific polymerase chain reaction (MSP-PCR) and restriction enzyme digestion to evaluate the methylation profile of *MMP-2* and *MMP-9* in 12 ameloblastoma samples and 12 healthy gingiva fragments, which were included as controls. Furthermore, we investigated the transcription levels of the genes by quantitative reverse-transcription PCR (qRT-PCR). Zymography was performed to verify protein expression in ameloblastomas.

**Results:**

The ameloblastomas showed a high frequency of unmethylated *MMP-2* and *MMP-9*, whereas the healthy gingival samples presented a sharp prevalence of methylated MMPs. Higher expression levels of *MMP-9* were found in ameloblastomas compared to healthy gingiva. However, no significant differences in the *MMP-2* mRNA expression between groups was found. All ameloblastomas showed positive expression of MMP-2 and MMP-9 proteins.

**Conclusions:**

Our findings suggest that expression of *MMP-9* is increased in ameloblastomas and is possibly modulated by unmethylation of the gene.

## Background

An ameloblastoma is a benign odontogenic tumour that exhibits a high recurrence risk, aggressive behaviour and local invasiveness [1]. Histologically, an ameloblastoma consists of epithelial strands or islands of ameloblastic epithelium. The peripheral cells are columnar, while the cells lying more centrally are fusiform to polyhedral and are loosely connected to each other [1].

Different studies have demonstrated genetic alterations in odontogenic tumours [2-4], but few studies have analysed epigenetic events in these tumours [5-7]. Methylation is an epigenetic alteration that plays an important role in controlling gene activity, embryonic development, and genomic imprinting. It has been associated with gene silencing by transcriptional inactivation [8]. DNA methylation or hypomethylation of the *p16p21* and *LINE-1* genes was reported in ameloblastomas by our group and others [5,9,10], but the significance of this data remains to be determined.

Matrix metalloproteinases (MMPs) are zinc-dependent enzymes that are important in extracellular matrix remodelling and are associated with tumour growth and invasion through collagen matrix degradation [11]. The invasive characteristic of ameloblastomas has been associated with the expression of genes related to bone turnover and extracellular matrix remodelling; these include *BMPRANKL* and its receptor, *MMP* and *TIMP*[12-17]. As MMPs may be regulated by DNA methylation in malignant neoplasms [18,19], such phenomenon might be important in ameloblastoma pathogenesis and should be investigated. Therefore, the purpose of this study was to investigate the association between *MMP-2* and *MMP-9* methylation and their mRNA transcription and protein expression in ameloblastomas.

## Methods

### Patients and tissue samples

Twelve fresh ameloblastoma specimens were collected during surgical care in the Department of Oral Surgery and Pathology, Universidade Federal de Minas Gerais, Brazil. These samples comprised eleven solid-multicystic follicular ameloblastomas and one unicystic case. Diagnoses were confirmed by histopathologic analysis based on the World Health Organization classification of histological typing of odontogenic tumours [1]. Other clinical data are shown in Table 1. Twelve fragments of healthy gingival samples with no clinical evidence of inflammation were collected during third molar extractions and used as controls. The samples were obtained following informed consent and with the approval of the *Universidade Federal de Minas Gerais* Ethics Committee (reference number 266/11). 

**Table 1:** Distribution of subjects according to gender, age and anatomic site

**Parameters**	**Ameloblastoma (n = 12)**	**Healthy gingiva (n = 12)**
**Gender**		
Male	05 (41.7%)	07 (58.3%)
Female	07 (58.3%)	05 (41.7%)
**Age (years)**		
Variation	8-51	19-28
Mean ± SD	31.0 ± 14.6	25.4 ± 5.3
**Anatomic Sites**		
Mandible		
anterior region	01 (8.3%)	0
posterior region	11 (91.7%)	4 (33.3%)
Maxilla	0	8 (66.7%)

### DNA isolation and methylation analysis of *MMP-2* and *MMP-9*

Genomic DNA was isolated from the tissue samples using a Qiagen DNeasy Tissue Kit (Qiagen Inc., Valencia, CA, USA) according to the manufacturer’s instructions. *MethPrimer* software [20] was used to search CpG islands and sparse CG dinucleotides. Distinct methods are suggested to analyse methylation profiles according to the presence of CpG islands or sparse CG dinucleotides located in the promoter region or in exons near to that region [21].

To assess the *MMP-2* gene CpG island methylation, genomic DNA was modified by sodium bisulfite as described previously [6] and subsequently amplified with primer sets designed to specifically recognise methylated (F 5’-GCGGTTATACGTATCGAGTTAGC-3’ and R 5’-ACTCTTTATCCGTTTTAAAAACGAC-3’; 205 bp) and unmethylated DNA (F 5’-GGTGGTTATATGTATTGAGTTAGTGA-3’ and R 5’-ACTCTTTATCCATTTTAAAAACAAC-3’ 206 bp). Bisulfite-treated unmethylated DNA from (peripheral blood mononuclear cells) cells was used as a positive control for unmethylated amplification of the *MMP-2* gene. Methylation-induced DNA of same cells by the MSssI methylase enzyme (New England Biolabs, Beverly, USA) was used as positive control for methylated amplification.

The methylation-sensitive restriction enzymes HhaI and AciI (New England BioLabs, Beverly, MA, USA) were used to assess the methylation of CG dinucleotides in the *MMP-9* promoter, including the CG sites located at positions -35, -185, -223, -233, as described previously [21]. Restriction enzymes cleave DNA at unmethylated CG sites, but they are unable to cut methylated cytosines. Analysis using a bioinformatics web site ( http://www.restrictionmapper.org) showed that the HhaI enzyme cleaves the restriction site at position -35 and that the other sites are cleaved by AciI. The CG dinucleotides analysed in this study are located close to the transcription start of the *MMP-9* gene. Two hundred nanograms of genomic DNA was digested separately with each of the restriction enzymes HhaI and AciI according to manufacturer's protocol to cleave the specific regions containing CG sites (New England BioLabs, Beverly, MA, USA). Digestion was followed by PCR amplification (primers: F 5’-GCTTCATCCCCCTCCCTCC-3’, R 5’-AGCACCAGGACCAGGGGC-3’; 369 bp). PCR products were subjected to electrophoresis in 6.5% polyacrylamide gels. While methylated cytosine produces a band equivalent to that of control methylated DNA of placenta tissue, the cleavage by restriction enzyme at unmethylated CpG induces DNA strand breaks, and thus no band is detected. In each PCR reaction, undigested DNA of each sample was also carried out as controls. Undigested and digested PCR products were electrophoresed in parallel. Human unmethylated DNA (Qiagen Inc., Valencia, CA, USA), which is sensitive to action of the enzyme, was also used as unmethylated positive control.

### RNA extraction and Quantitative Real-time PCR (qRT-PCR) of *MMP-2* and *MMP-9*

Total RNA was extracted from tissue samples using Trizol reagent according to the manufacturer’s protocol (Invitrogen, Carlsbad, CA, USA). RNA integrity was analysed by 1% agarose gel electrophoresis. Reverse transcription of 1 μg of RNA to cDNA was performed using SuperScript III First-Strand (Invitrogen, Carlsbad, CA, USA) following the manufacturer’s instructions. Primer sequences were designed using the *PrimerExpress* software (v.3, Applied Biosystems) as follows: *MMP-2* (F: 5’-AGCTCCCGGAAAAGATTGATG-3’; R: 5’-CAGGGTGCTGGCTGAGTAGAT-3’, 101 bp) and *MMP-9* (F: 5’-GAGGTTCGACGTGAAGGCGCAGAT-3’; R: 5’-CATAGGTCACGTAGCCCACT TGGT3’, 200 bp). All reactions were run in duplicate in a StepOne Real time PCR System using the SYBR-green fluorescence quantification system (Applied Biosystems, Warrington, UK). The comparative *C*_*t*_ method was used [22]. Expression levels of the *MMP-2* and *MMP-9* genes relative to a calibrator sample (placenta tissue) were obtained by normalisation to endogenous *β-actin*.

### Gelatin zymography

Ameloblastoma protein was extracted and subjected to electrophoresis under nonreducing conditions on SDS-polyacrylamide gels copolymerised with 1 mg/ml gelatin (Sigma Chemical Co, St Louis, MO, USA) as previously described [23]. After electrophoresis, the gels were washed in 2.5% Triton-X 100 and incubated for at least 18 h at 37°C in incubation buffer (50 mM Tris–HCl, pH 7.5, containing 5 mM CaCl_2_, 100 mM NaCl, 0.01% Triton X-100). Zymographic gels were stained in 0.2% Coomassie Brilliant Blue R-250 and de-stained. The gels were scanned to analyse the bands representative of MMPs, according to molecular weight. Analysis of protein expression in healthy gingiva was not performed due to the scarcity of tissue samples.

### Statistical analysis

Mann–Whitney tests were used to compare the relative quantification of *MMP-2* and *MMP-9* between groups. Chi-squared or Fisher’s exact were used when appropriate. The analyses were carried out using SPSS 17.0 software, and probability values <0.05 were considered statistically significant.

## Results

**Figure 1:**
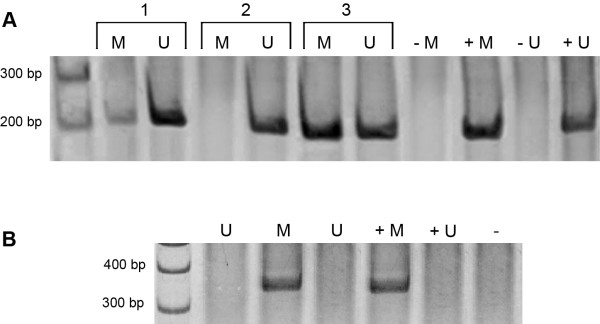
**Representative figure of the methylation analysis.A**: Methylation status of *MMP-2* in ameloblastoma. M: PCR products when amplified by methylated primers (205 bp); U: PCR products when amplified by unmethylated primers (206 bp); +M: positive control for methylated reaction; +U: positive control for unmethylated reaction. -M and -U: negative controls without DNA. Lines 1 to 3 represent DNA from ameloblastoma samples. **B**: Methylation status of *MMP-9* in ameloblastoma. DNA samples were digested by the AciI restriction enzyme followed by PCR, flanking the restriction sites. Absent band indicates unmethylated profile (U) due to DNA cleavage by the restriction enzyme. Presence of the PCR band represents methylated profile (M) of the *MMP-9* gene. +M: methylated positive control; +U: unmethylated positive control; - : negative control without DNA.

*MMP-2* and *MMP-9* methylation statuses are shown in Table 2 and represented in Figure 1. While all healthy gingival samples showed *MMP-2* methylation, approximately half of ameloblastomas were unmethylated. Similarly, an increased frequency of unmethylated *MMP-9* of specific CG sites digested by HhaI was identified in the ameloblastomas. Almost all of the ameloblastoma samples showed an unmethylated profile for *MMP-9*. No difference was found in the methylation of CG sites digested by Acil among the groups studied.

**Table 2:** **Methylation status of *MMP-2 *and *MMP-9 *genes in ameloblastoma and healthy gingiva**

**Parameters**	**Ameloblastoma (n = 12)**	**Healthy gingiva (n = 12)**	p-value*
**MMP-2 methylation**			
Methylated	05 (41.7%)	12 (100.0%)	0.002*
Unmethylated	07 (58.3%)	0 (0.0%)	
**MMP-9 methylation/restriction site digested by HhaI**			
Methylated	0 (0.0%)	06 (50.0%)	0.006*
Unmethylated	12 (100.0%)	06 (50.0%)	
**MMP-9 methylation/restriction site digested by AciI**			
Methylated	01 (8.3%)	0 (0.0%)	0.317
Unmethylated	11 (91.7%)	12 (100.0%)	

* Fisher’s exact test (p < 0.05).

**Figure 2:**
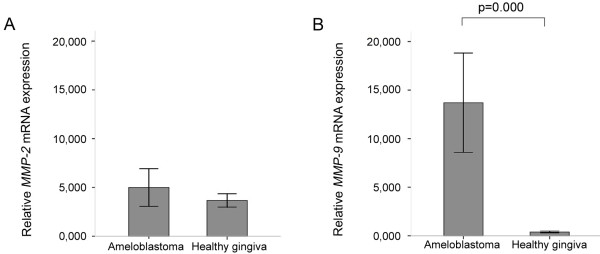
**qRT-PCR results of *MMP-2 *(A) and *MMP-9 *(B) mRNA transcription in ameloblastoma and healthy gingiva.** In both analyses, gene expression is shown as the mean ± SE. All reactions were normalised to β-actin and are relative to a calibrator sample. * Mann–Whitney test (p < 0.05).

The qRT-PCR results are summarised in Figures 2a and 2b. Higher expression levels of *MMP-9* were found in ameloblastomas compared to healthy gingiva. However, significant differences in the *MMP-2* mRNA expression levels were not found.

When we investigated the influence of the methylation status of both genes on their transcription, no association was found between *MMP-2* transcription and its methylation in ameloblastomas (p = 0.319). Almost all of the tumour samples showed an unmethylated *MMP-9* pattern in conjunction with increased mRNA levels. As most of the ameloblastomas were unmethylated at the *MMP-9* gene, considering all of the restriction sites, it was not possible to statistically compare the transcription of the gene in the cases with or without methylated sequences.

**Figure 3:**
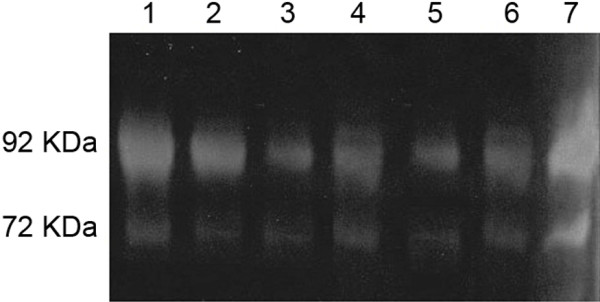
**Zymography gel electrophoresis representing MMP-2 (72 KDa) and MMP-9 protein (92 KDa) in ameloblastoma.** Lines 1 to 7 represent protein extracts from ameloblastoma samples.

All of the ameloblastoma samples showed expression of MMP-2 and MMP-9 proteins, as verified by zymography (Figure 3). However, pro-MMP-2 and pro-MMP-9 forms were not identified in ameloblastomas.

## Discussion

The underlying molecular pathways associated with the pathogenesis of ameloblastomas are not well established yet. Previous investigations have assessed the molecular and genetic alterations related mainly to apoptosis, allelic loss of tumour suppressor genes, deregulation of the Sonic Hedgehog signalling pathway, and the clonality of these tumours [3,24,25].

Matrix metalloproteinases are involved in the degradation of collagen, as well as bone matrix, and have been shown to play a key role in the local invasiveness of ameloblastoma cells [15,26]. Overexpression of *MMP-2* and *MMP-9* was associated with the infiltrative behaviour of ameloblastomas, as well as that of several malignant neoplasms [17,27]. The suppression of *MMP-2* activity was able to inhibit the invasiveness of ameloblastoma cells *in vitro*[14,15]. Furthermore, it was suggested that increased expression of *MMP-9* may be involved in the proliferation and invasive behaviour of ameloblastomas [26].

Some papers, including studies from our research group, have demonstrated epigenetic alterations in odontogenic tumours [5,6,9,10,28]. In the present study, we hypothesised that methylation may regulate the expression of *MMP-2* and *MMP-9* in ameloblastomas. We also investigated the association between methylation and the transcription levels of these genes. As most of the ameloblastoma samples were of the solid follicular type, we were not able to analyse possible associations between each clinical or histological type and the molecular data.

MMPs play an important role in collagen matrix remodelling in physiologic and pathologic processes, such as those found in basal membranes, dental follicle tissue and tumour metastasis [27,29]. Although *MMP-2* is related to ameloblastoma pathogenesis, it seems to be constitutively expressed in physiologic tissues and many cell types and to exhibit characteristics of a housekeeping gene [30-32]. Perhaps this could explain the similar expression levels of *MMP-2* mRNA in ameloblastomas and healthy gingiva. Furthermore, our data suggest that *MMP-2* expression in ameloblastomas may not be modulated by methylation because the methylation profile for this gene did not correlate with *MMP-2* transcript levels in this odontogenic tumour.

The ameloblastomas presented an unmethylated profile of *MMP-2* and *MMP-9* genes compared to gingiva. Furthermore, along with unmethylated *MMP-9*, this tumour showed increased transcription of *MMP-9* when compared to the control group. The important role of methylation in epigenetic silencing is well established, particularly through regulatory mechanisms of transcription. Accordingly, our data suggest that an unmethylated profile of the *MMP-9* gene in ameloblastomas may be a permissive event allowing the binding of transcription factors to DNA, thus favouring *MMP-9* gene transcription.

All of the ameloblastomas showed MMP-9 protein expression and were mostly unmethylated for *MMP-9*, so it was not possible to assess if the transcription of the gene was correlated with its methylation status. However, our study suggests that the increased transcription of *MMP-9* in ameloblastomas could possibly be influenced by unmethylation of the gene. The evident protein expression, identified by zymography, provides additional evidence supporting the possible gene regulation by unmethylated *MMP-9*. It is interesting to note that hypomethylation of the *MMP-2* and *MMP-9* genes increases gene expression and contributes to cancer cell invasiveness and tumourigenesis in malignant neoplasms, such as prostate cancer and lymphoma [18,33].

## Conclusion

In conclusion, our study provides new insights into the epigenetic regulation of MMPs in ameloblastomas and points to the hypomethylation of *MMP-9* as a possible mechanism involved in the increased transcription of the gene in this tumour. However, functional studies are needed to better explain the role the methylation of matrix metalloproteinases plays in the pathogenesis of ameloblastoma.

## Competing interests

The authors declare that they have no competing interests.

## Authors’ contributions

RSG, CCG, and LCF participated in the study design and drafted the manuscript. LCF and MCS performed the experiments. EFF performed the statistical analysis. WHC and JCTL collected the ameloblastoma samples and clinical information. RSG and CCG reviewed all of the histological diagnoses. All of the authors read and approved the final manuscript.

## Pre-publication history

The pre-publication history for this paper can be accessed here:

http://www.biomedcentral.com/1472-6890/12/11/prepub
